# Inhibition of Soluble Epoxide Hydrolase Prevents Docetaxel-Induced Painful Peripheral Neuropathy

**DOI:** 10.3390/ijms26125630

**Published:** 2025-06-12

**Authors:** Karen M. Wagner, Jun Yang, Christophe Morisseau, Bruce D. Hammock

**Affiliations:** Department of Entomology and Nematology and UC Davis Comprehensive Cancer Center, University of California Davis, Davis, CA 95616, USA; kmwagner@ucdavis.edu (K.M.W.); junyang@ucdavis.edu (J.Y.); chmorisseau@ucdavis.edu (C.M.)

**Keywords:** docetaxel, chemotherapy-induced peripheral neuropathy, soluble epoxide hydrolase, pain, nociception, analgesics

## Abstract

Chemotherapy-induced peripheral neuropathy (CIPN) is a painful condition recalcitrant to current available therapies. CIPN pain can be severe and dose-limiting or dose-reducing for life-extending chemotherapeutics and, to date, there is no treatment to alter the progression of CIPN. For these experiments we used docetaxel, a first-line therapy for metastatic prostate cancer in humans and investigated the soluble epoxide hydrolase inhibitor EC5026 for its analgesic efficacy against this CIPN pain. Male SD rats (n = 10/group) were pretreated with 1 mg/kg EC5026 in formulated drinking water or vehicle for one week prior to docetaxel injections. The rats continued the formulated drinking water during three once-a-week docetaxel 10 mg/kg i.p. injections and were maintained on treatment until the end of week 5 when all groups were transitioned to normal drinking water. Nociceptive testing occurred throughout the entire experiment including after transitioning to normal drinking water. EC5026 increased mechanical withdrawal thresholds and latencies on the cold plate compared to docetaxel-treated controls. There were no motor effects of the compound, and the formulated drinking water provided favorable exposure. These results demonstrated that EC5026 administered prophylactically was both analgesic and able to limit the severity of mechanical and cold sensitivities in the docetaxel CIPN rat model.

## 1. Introduction

Cancer patients receiving chemotherapeutics suffer neurotoxicity as one of the side effects of their life-saving antineoplastic treatments [1]. Chemotherapy-induced peripheral neuropathy (CIPN) is a painful condition, with taxanes being one of three classes of agents well established in causing CIPN as a side effect of treatment [2,3]. A recent survey revealed that the majority (63%) of chemotherapy-treated patients suffer from painful CIPN, with over 30% receiving dose reductions or discontinuation due to their symptoms and at least 40% reporting persistent pain following treatment [4]. Prostate cancer specifically is the second leading cancer in men globally [5], is a leading cause of cancer-related mortality in men worldwide [6] and tops the list at 27% of new cancer diagnoses in males [7]. Docetaxel is a leading chemotherapeutic agent to combat metastatic castration-resistant prostate cancer [8]. Docetaxel is a microtubule-stabilizing taxane that blocks the cell cycle, limiting the progression of cancer [9]. However, the same mechanism of action is also responsible for the neurotoxic action that leads to painful peripheral neuropathy. Taxanes bind polymerized tubulins but also bind mitochondrial β-tubulin that can cause damage by opening the mitochondrial permeability transition pore [10]. To date, there is no available therapy known to prevent CIPN [11]. It has been predicted that there will be a surge in new prostate cancer cases worldwide by 2040 [12]. With increased cancer survival rates, there is increased importance of the lingering deleterious effects of chemotherapy, including the chronic pain syndrome that persists and cognitive impairments after chemotherapy.

The analgesic potential of inhibiting soluble epoxide hydrolase (sEH) has been described in preclinical studies using the three classes of chemotherapeutics known to cause CIPN [13]. sEH is a regulator of endogenous epoxy fatty acids (EpFAs) which are lipid signaling molecules that arise from the metabolism of several classes of long-chain polyunsaturated fatty acids (PUFAs) [14]. EpFAs are normally short-lived eicosanoids formed by cytochrome P450’s action on parent PUFAs at any of their double bonds. The bioactive EpFAs are transformed by sEH into vicinal diols per each regioisomer. Inhibition of sEH extends the residence time of EpFAs and consequently their bioactivity. EpFAs have long been demonstrated to be analgesic, acting on inflammatory pain [15,16,17] as well as chronic pain conditions [18,19,20], including demonstrated efficacy against CIPN chronic pain specifically [13]. The mechanisms of sEH inhibition and EpFAs include limiting endoplasmic reticulum stress (ER stress) and mitochondrial dysfunction [21,22,23], and these mechanisms are known to contribute to the development of CIPN [24,25,26,27]. This suggests potential for sEH inhibitors to limit the development or progression of neurotoxicity and painful CIPN as well as therapeutically treat it. Thus, this hypothesis was tested here with pretreatment of the sEH inhibitor EC5026 formulated in drinking water to combat docetaxel-induced CIPN pain in non-tumor-bearing rats.

## 2. Results

### 2.1. EC5026 Effects on Mechanical Allodynia

**Figure 1:**
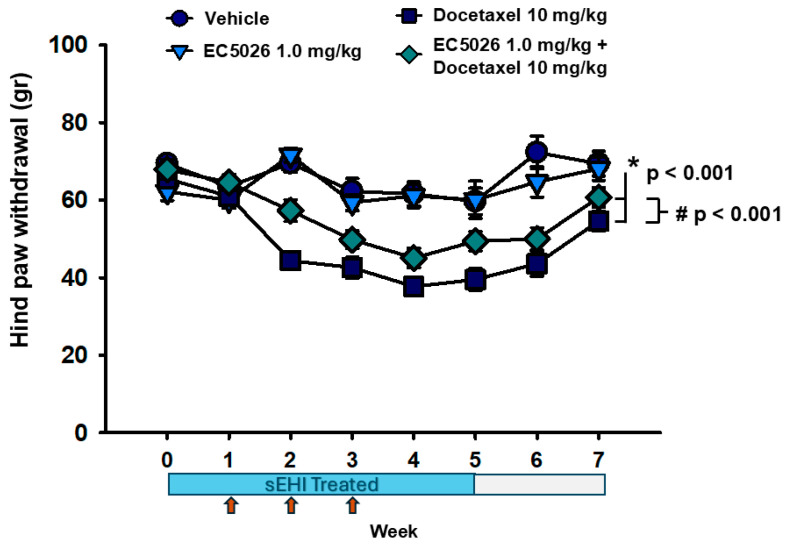
Prophylactic and continued administration of EC5026 limits the severity of chemotherapy-induced neuropathic pain.

In the CIPN model, docetaxel induced significant decreases in the von Frey assay responses (Figure 1) compared to both vehicle and EC5026 controls (* *p* < 0.001). Despite the overall decreases for docetaxel-treated groups, the prophylactic administration of the sEHI EC5026 increased the von Frey scores (# *p* < 0.001) compared to docetaxel alone over the course of the experiment. Importantly, these increases were sustained even after removal of the sEHI treatment and return to normal drinking water (weeks 6 and 7), indicating that these changes were more than compound-related (one-way repeated-measure analysis of variance, Tukey test post hoc analysis, n = 10/group).

Rats (n = 10/group) with docetaxel-induced CIPN and controls were assayed for mechanical allodynia in a von Frey assay that demonstrated the prophylactic effect of EC5026 delivered in formulated drinking water. The rats were pretreated with EC5026 targeting a 1 mg/kg dose or vehicle control for one week prior to and during three weekly docetaxel injections plus one additional week before transitioning to normal drinking water. The weekly docetaxel injections (red arrows) significantly lowered von Frey scores (* *p* < 0.001) and the EC5026 treatment significantly (# *p* < 0.001) increased withdrawal thresholds compared to docetaxel controls over the entire time course. EC5026 administration did not completely alleviate the CIPN sensitivity but was analgesic, and notably, the improvements were observable even after the cessation of sEH inhibitor, indicating a durable improvement in docetaxel-treated animals.

### 2.2. Cold Pain Response and Blood Concentrations of Ec5026

**Figure 2:**
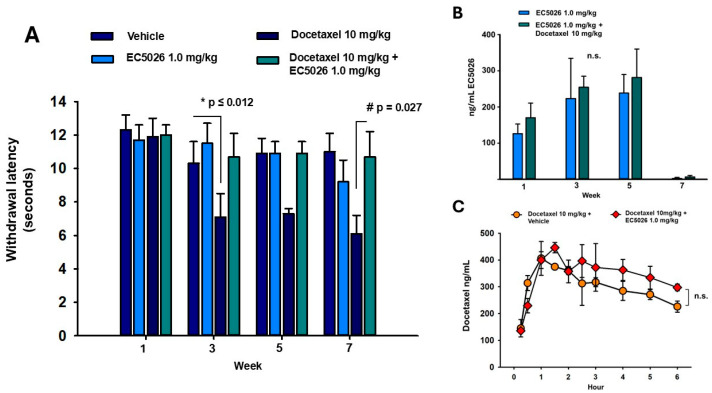
EC5026 is effective against cold-induced pain in the docetaxel CIPN model, and the efficacy remains after transition to normal drinking water. (**A**) In rats (n = 10/group), pretreatment of EC5026 (Week 1) had no effect on the responses in the cold plate assay. The docetaxel treatment resulted in significantly shorter latencies on the cold plate over the course of the treatment compared to controls (* *p* ≤ 0.012 for both controls). EC5026 formulated in the drinking water was able to improve the latencies of docetaxel treated rats on the cold plate (# *p* = 0.027) which persisted after their return to normal drinking water (Week 7). (**B**) The EC5026 formulated drinking water resulted in effective concentrations of EC5026 in whole blood. Blood concentrations of EC5026 were determined in a subset (n = 5/group) of both the EC5026 control and EC5026 + docetaxel-treated groups that received the compound formulated in 1% PEG400 drinking water. EC5026 administration began at the start of week one prior to docetaxel injections. The docetaxel was then dosed in three weekly injections and the drinking water maintained up to Week 5 when the rats were transitioned to normal drinking water though to the end of Week 7. The results indicate that there was good coverage at the beginning of docetaxel treatments and continued to increase but remained steady over the remainder of the experiment. The Week 7 values demonstrate the washout of the compound and that behavioral assay results were independent of EC5026 blood concentration. The EC5026 concentration was also not affected by docetaxel administration over the course of the experiment with no significance (n.s., *p* = 0.482) between the groups and animals continued to consume sufficient formulated drinking water despite the effects of the CIPN model. (**C**) In a separate acute PK experiment with pretreatment of EC5026 and a single docetaxel injection, there was no significant difference (n.s., *p* = 0.469) in the docetaxel concentration between the vehicle or EC5026 treated animals (n = 3/group).

The cold plate was set to 5 °C due to the neuropathy in treated animals and all groups were assessed at this temperature. Responses to cold plate stimulus (Figure 2A) revealed a decline in latency with docetaxel treatment compared to controls that remained post treatment to week 7. The docetaxel treatment significantly reduced the latency on the cold plate compared to vehicle (*p* = 0.006) and EC5026 (*p* = 0.012) controls. EC5026 in docetaxel-treated rats was able to reverse this decline (*p* = 0.027) during the course of docetaxel (weeks 1–3) and almost completely alleviated it for the residual neuropathy at weeks 5–7 (two-way repeated-measure analysis of variance, pairwise Holm–Sidak method post hoc analysis, n = 10/group).

EC5026 concentration was determined in whole blood samples by tail venipuncture in a subset of treated animals with the drinking water (Figure 2B). Blood was sampled for a pretreatment baseline and then once a week for the course of the experiment. The formulated drinking water was administered one week prior to the beginning of docetaxel injections, which lasted for 3 weeks, and continued to week 5 after which animals were transitioned to normal drinking water until the end of the experiment. The targeted dose was 1 mg/kg/day and consumption was assumed to be 10 mL water/100 gr body weight. This administration resulted in 250–280 ng/mL or 0.6–0.7 µM of EC5026, which has a Ki for the human enzyme of <0.05 nM [28], and that of the rat enzyme IC50 is estimated to be 1.4 nM (Table A1). Importantly, there was no statistical difference between the EC5026 + docetaxel and EC5026 control groups (*p* = 0.481), indicating there was no effect of the docetaxel on the concentration of the sEH inhibitor and that the formulated inhibitor was well tolerated and consumed to meet a blood concentration of favorable exposure (two-way repeated-measure analysis of variance, Holm–Sidak method post hoc analysis, n = 5/group). To more adequately investigate the acute effects of EC5026 and docetaxel coadminstration we assessed the concentrations of both compounds in a shorter paradigm. For this, rats were repeatedly oral gavaged (daily, on four consecutive days) with either 1 mg/kg EC5026 in 100% PEG400 or the vehicle and received one dose of 10 mg/kg docetaxel i.p. and then were followed over a time course to examine the blood concentration of docetaxel. The analysis revealed that there was no significant difference (*p* = 0.469) in the docetaxel concentration between the vehicle or EC5026 treated animals. Thus, there is no effect of EC5026 altering docetaxel concentrations. (two-way repeated-measure analysis of variance, Holm–Sidak method post hoc analysis, n = 3/group).

### 2.3. Open-Field and Grip Strength Functional Assays

**Figure 3:**
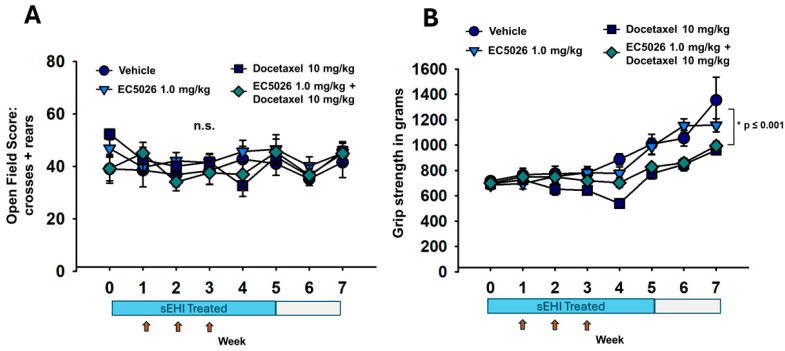
All groups of rats demonstrated motor skills and coordination throughout the experiment. (**A**) The rats (n = 10/group) were assessed with the open-field assay weekly during the course of the experiment (red arrows indicate docetaxel injections) to evaluate any potential changes in motor skill or self-motivated movement/exploration. The rats in all groups performed well with no significant differences between groups (n.s., *p* = 0.880). (**B**) The rats (n = 10/group) only began to display changes in grip strength over the course of docetaxel injections (red arrows) when the docetaxel control and EC5026-treated docetaxel groups scored lower in the assay compared to untreated controls. This was a significant difference (* *p* < 0.001) and remained over the duration of the experiment. The grip strength increased over time concurrently with increases in rat body weight, and despite the docetaxel groups scoring lower compared to controls, their scores maintained an increase with growth over the duration of the experiment.

The open-field assay (Figure 3A) demonstrated there was no change in the exploration or motor skill of animals treated with docetaxel (either group) or the EC5026-treated control group over the course of the experiments. This functional assay demonstrates that the animals were not incapacitated by the docetaxel treatments and thereby supports the results in nociceptive assays. It also agrees with previously published data on administration of sEHI inhibitors, which have not been observed to alter gaiting or motor skills while eliciting their analgesia [13,29] (*p* = 0.880, two-way repeated-measure analysis of variance, Holm–Sidak method post hoc analysis, n = 10/group). The grip strength assay (Figure 3B) demonstrated that there was some loss of strength in the docetaxel-related groups compared to controls (* *p* < 0.001) but not between the EC5026-treated docetaxel group compared to the docetaxel control (*p* = 0.223). The apparent increase in grip strength over the seven-week course of the experiment correlates with the increased body weight and age of the rats (Friedman repeated-measure analysis of variance on ranks, Tukey Test post hoc analysis, n = 10/group).

### 2.4. Body Weight Comparisons

**Figure 4:**
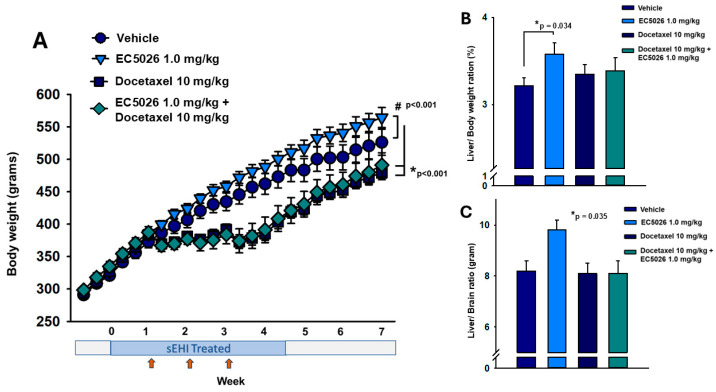
EC5026 did not alter the weight gain suppression due to docetaxel treatment. (**A**) The body weight of all rats (n = 10/group) was assessed every 2 days during the experiment. The results revealed a suppression of weight gain with docetaxel treatment which resumed after the course of 3 weekly injections (red arrows) but never reached the level of controls. Both the vehicle and the EC5026 controls were statistically (* *p* < 0.001) increased over the docetaxel control and the EC5026 docetaxel treated rats. The two docetaxel treated groups showed no statistically significant change when compared, however, the EC0526 control group was significantly increased (# *p* < 0.001) compared to the vehicle control group. The CNS tissues and major organs including whole liver were weighed at sampling for all animals included in the experiments. (**B**) The comparison of liver-to body weight among the groups (n = 10/group) revealed a significant increase in liver weight for the EC5026 control group compared to the vehicle control (* *p* = 0.034). (**C**) Due to the significant body weight loss in some groups, all the liver weights (n = 10/group) were then normalized to the brain weight for each individual animal and confirmed a statistically significant difference between the groups (* *p* = 0.035).

The body weight of the rats was monitored every two days to ensure the rats were not suffering pernicious weight loss from the chemotherapy regimen. This data revealed that the docetaxel treatment suppressed weight gain (* *p* <0.001) in chemotherapy-treated animals (Figure 4A). Once the three weekly injections of docetaxel were complete, these rats resumed weight gain but remained behind the control-treated animals. The EC5026 treatment failed to alter this docetaxel-induced suppression of weight gain and there was no observable difference in weight between the two groups treated with docetaxel (*p* = 0.999), with the EC5026-treated docetaxel group also significantly lower than both controls (* *p* < 0.001). Interestingly, the EC5026 control group (no docetaxel) increased in weight over the duration of the experiment compared to vehicle controls (# *p* < 0.001) (Friedman repeated-measure analysis of variance on ranks, Chi-square = 196.6 with three degrees of freedom, Tukey test post hoc analysis, n = 10/group). Liver weights were measured at necropsy and a statistically relevant increase was seen in the EC5026 control group per gram of whole liver (one-way analysis of variance, Holm–Sidak post hoc analysis, *p* = 0.019, n = 10/group). An analysis of the percent of liver-to-body weight ratio (Figure 4B) resulted in no significant difference (Kruskal–Wallis one-way analysis of variance on ranks, n = 10/group, *p* = 0.164). We also compared the liver-to-body weight ratios between vehicle- and EC5026-treated controls rats separately avoiding non-normally distributed data and this revealed the EC5026-treated rats had a statistically significant increase (* *p* = 0.034) in the ratio compared to vehicle control rats (*t*-test, *t* = −2.299 with 18 degrees of freedom, two-tailed). While it has been previously reported that the liver-to-body weight ratio for male rats has the best fit in healthy control animals, in the case of the docetaxel CIPN model where weight gain was suspended during the chemotherapy treatment, it is possible that using the body weight as the denominator could result in an ‘artifactual change’ in the relative weight of the liver [30]. We therefore analyzed the liver-to-brain weight because, per Sellers et al., in cases of notable changes in body weight, tested materials do not often change brain weights [31]. This liver-to-brain ratio (Figure 4C) revealed an overall statistically significant difference in a pairwise comparison of the included groups (F = 3.186, * *p* = 0.035); however, the post hoc analysis did not identify specific pairwise differences (one-way analysis of variance, n = 10/group, Holm–Sidak post hoc analysis).

### 2.5. ELISA Quantification of sEH Protein in Tissue

**Figure 5:**
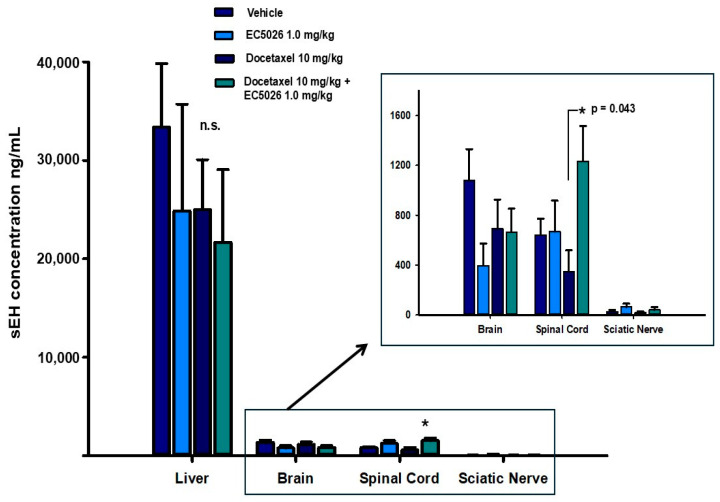
Docetaxel treatment and induced CIPN did not increase the concentration of the sEH protein measured by ELISA 4 weeks after the end of treatment. Docetaxel did not elevate the concentration of sEH protein in liver per the samples collected at the end of the seventh week (n.s., *p* = 0.343) (n = 6 docetaxel and EC5026 groups, n = 8 vehicle and EC5026 + docetaxel groups). Insert: All the analyzed CNS tissues from the same animals were low when compared to liver concentrations. In addition, the relative amount of sEH in the sciatic nerves was low when compared to the other CNS tissues. The spinal cord showed a statistically significant increase specifically in the EC5026 + docetaxel group compared to the docetaxel control (* *p* = 0.043), however, there were no other changes in sEH protein among the treatment groups or included tissues. This is despite the docetaxel treated rats exhibiting increased sensitivity in the nociceptive assays at this time point.

An enzyme-linked immunosorbent assay (ELISA) for sEH was used to quantify the protein from the animals that were assessed for nociceptive outcomes (Figure 5). These tissues were sampled at the end of week seven after completion of the behavioral experiments. This was 4 weeks post the last docetaxel injection and 2 weeks post the end of EC5026 formulated drinking water. In the nociceptive assays, the docetaxel control group was still exhibiting lower thresholds and latencies in the nociceptive assays, indicating persistent painful neuropathy at the time of euthanasia. The mean ± SEM per group results revealed there was no significant change in the liver concentrations of the sEH protein among the four included groups (*p* = 0.343, one-way analysis of variance, n = 6 for docetaxel and EC5026 groups, n = 8 for vehicle and EC5026 + docetaxel groups). There was also no change in the brain (*p* = 0.257) or sciatic nerves (*p* = 0.262) among the four treatment groups (insert) (Kruskal–Wallis one-way analysis of variance on ranks per tissue, n = 6 for docetaxel and EC5026 groups, n = 8 for vehicle and EC5026 + docetaxel groups). The spinal cords displayed only statistical differences in the tissues, with the comparison of the EC5026 docetaxel group having a statistically significant increase (* *p* = 0.043) over the docetaxel group but not the vehicle or EC5026 controls (insert) (one-way analysis of variance, Holm–Sidak method post hoc analysis, n = 6 for docetaxel and EC5026 groups, n = 8 for vehicle and EC5026 + docetaxel groups). Additionally, the results indicated that the liver had comparatively the highest concentration of sEH protein, as expected, and there was a far lower sEH concentration in the nervous system tissues (insert), including the brain and spinal cord, with sciatic nerves having the lowest overall compared to the liver.

## 3. Discussion

**Figure A1:**
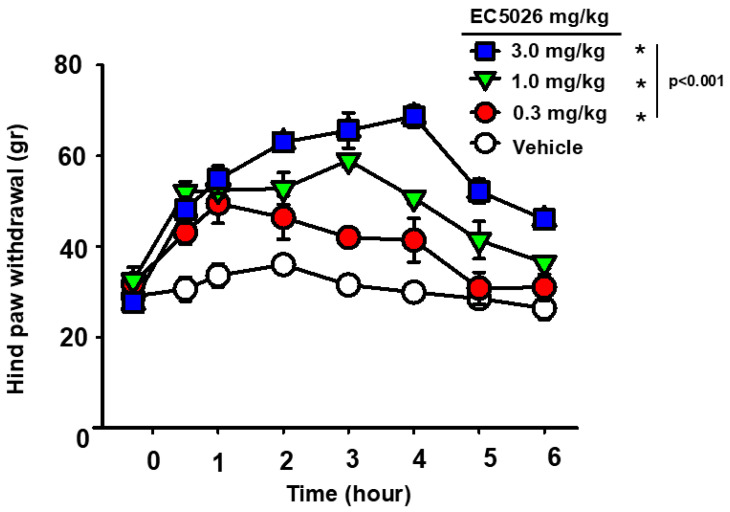
Therapeutic treatment of docetaxel-induced CIPN in male rats with EC5026 reveals dose-dependent analgesia.

The aim of this study design was to determine if EC5026 was analgesic against docetaxel-induced CIPN and if the sEH inhibition could alter the progression of docetaxel-induced CIPN pain. Inhibiting sEH has demonstrated effects on limiting ER stress and mitochondrial dysfunction [21,22,23]. ER stress and mitochondrial dysfunction are hypothesized mechanisms of neuronal damage, and these mechanisms have been known to be involved in chronic pain conditions overall [32,33], as well as in CIPN specifically [24,25,26,34]. Rats were pretreated with EC5026, and the von Frey nociceptive assay was used to determine the mechanical sensitivity to an innocuous stimulus in the rats. This assay has face validity compared to the clinical use of monofilaments, which has high correlation to the WHO criterion for sensory changes in human CIPN patients [35,36]. A cold plate assay was also used because cold pain is one of the leading clinical adverse side effects experienced by patients with CIPN [37]. The pre- and concurrent treatment of EC5026 with the docetaxel injections did not completely prevent a decrease in the tactile allodynia or cold pain scores, but importantly, it did reduce the amount of sensitivity over the course of the treatment (Figure 1). Perhaps more remarkable was the fact that the EC5026-treated group maintained improvements over the CIPN group even after the rats were transitioned to normal drinking water. This indicates that the treatment was not only analgesic while on board but may have prevented some of the neuronal damage that contributes to the painful neuropathy. Previously, the same inhibitor, EC5026, was able to dynamically alter the von Frey paw withdrawal thresholds when dosed acutely against established painful paclitaxel CIPN in both female and male rats [13]. We therefore also investigated the acute treatment using the established pain of a docetaxel-induced CIPN model in male rats for comparison (Figure A1, [38,39]). In this therapeutic paradigm, EC5026 treatment revealed robust and dose-dependent analgesia in the docetaxel-induced CIPN model. It bears consideration that the effects of prophylactic and prolonged EC5026 seem less dynamic in direct comparison to the acute therapeutic paradigm, but it is known that habituation can produce a reduced response to repeated stimuli, and the more frequent the stimulation, the more pronounced the habituation [40]. The different experimental designs of the acute therapeutic administration following a one-day time course versus the prolonged prophylactic administration being tested every week for 7 weeks plays a role when comparing these outcomes head to head. Additionally, in the nine criteria recognized in forming a response habituation, it was identified that weaker stimuli may have more pronounced habituation, e.g., the von Frey threshold probe compared to the suprathreshold cold plate temperature [40]. Thus, although the behavioral experiments were scheduled and routinized to provide a consistent interval that avoided frequent stimulation with the assays over the duration of the 7-week experiment, the habituation to the assay stimulus was, to some extent, unavoidable in tracking the progress over this long a duration. Despite these factors, both this acute therapeutic strategy and the prolonged prophylactic treatment regimen improved the nociceptive outcomes, indicating analgesia. Notably, the longer-term treatment continued to be efficacious over the duration of the experiment, and the improved nociceptive assay scores persisted in the absence of the sEH inhibitor (weeks 5–7) in these experiments. More specific studies are needed to further investigate this outcome, but this initial result is promising because of the demonstrated halt in progression of CIPN pain measured with behavioral assays in whole animals.

Perhaps the most robust response and significant outcome was observed in the cold plate assay (Figure 2). It is well documented that there is a strong cold pain associated with oxaliplatin chemotherapy, but taxanes, including paclitaxel and docetaxel, induce cold allodynia as well [41,42]. In humans, cold pain thresholds have a wide range, even in healthy volunteers, with some non-responders even at 5 °C [43,44]. We used 5 °C in these experiments to ensure the safety of rats in blinded groups, which included animals with neuropathic pain sensitivity. Despite inherent variability in the cold response, the results from the cold plate assay indicate that EC5026 was able to almost completely prevent the decline in latency compared to docetaxel controls (Figure 2A). The use of a cold plate versus packed dry ice or acetone to assay cold pain in these experiments had the advantage of applying a controlled stimulus intensity, which was important in repeatedly assessing the evoked sensory responses. The apparent increased efficacy for this pain modality over the mechanical allodynia may be due to the less frequent repeated testing (fewer trials per average) as well as the intensity of the stimulus. As noted earlier, these criteria of frequency and stimulus intensity can impact habituation in longer-term experiments. Research on cold sensing mechanisms indicates different channel involvement such as transient receptor potential melastatin 8 (TRPM8), ankyrin 1 (TRPA1), and sodium channel subtype (Nav)1.8 [45] compared to sensing mechanical allodynia involving Nav1.7 and the potassium channel (Kv1.1) [46], but all these channels contribute to signaling in sensitized C and Aδ fiber nociceptors suffering mitochondrial toxicity and the ER stress effects of docetaxel. Thus, regardless of this difference, both pain modalities demonstrated improvements with EC5026 treatment in the CIPN rat model. There are known anti-inflammatory effects of sEH inhibition, and they have demonstrated analgesic efficacy against inflammatory pain [47,48]; however, it is known that anti-inflammatory drugs do not successfully block the pain of CIPN [49,50]. The success of EC5026 in blocking painful CIPN indicates that additional mechanisms contribute to its efficacy in this pathology.

**Figure A2:**
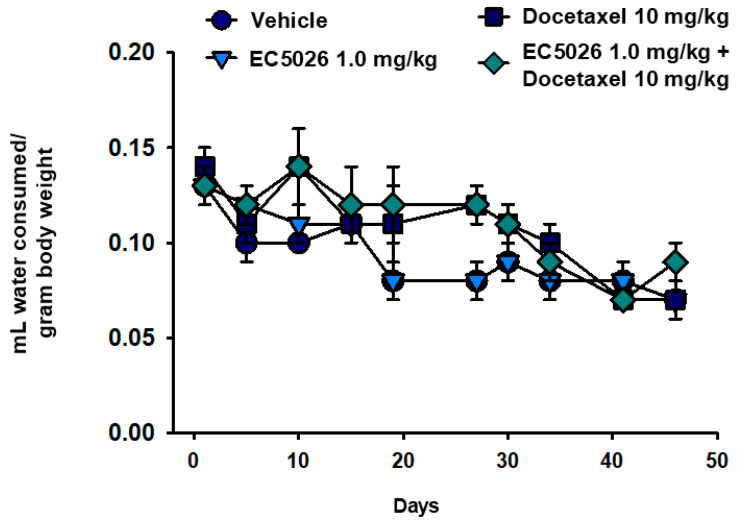
Consumption of drinking water was not affected by EC5026 formulation.

The analgesic efficacy correlated with favorable exposure of the sEHI in whole blood. EC5026 blood concentrations were monitored in a subset of animals throughout the experiment to examine the formulated drinking water consumption. The results indicated absorption of EC5026 in whole blood with no significant effect of the docetaxel treatments on the sEHI concentration (Figure 2B). The drinking water formulation enabled a continual daily dosing in the experiment without handling and procedural stress or the risk of injury of oral gavage or daily injections. The drinking water formulation was well tolerated and consumed in typical amounts (Figure A2). However, the experimental paradigm did not allow an evaluation of the effect EC5026 may have had on docetaxel concentrations. Therefore, we investigated the pharmacokinetics of acute coadministration of the compounds, and there was no change in the concentration of docetaxel in EC5026- vs. vehicle-treated rats (Figure 2C). Thus, neither compound impacted the pharmacokinetics of the other compound in this study.

The open-field assay (Figure 3A) was used once a week to ensure the motor skill and self-initiated movement in the animals, especially for those receiving docetaxel, which may cause substantial weight loss and is known to cause fatigue in humans. This assay showed no significant changes over the course of the experiment, which ensured that the animals were functionally able to perform in the nociceptive assays. With the blood concentrations measured at around 0.6–0.7 µM, the assay also identified EC5026 specifically among sEH inhibitors as a class of compounds that have been tested and shown to induce no alteration in motor skill despite their potent analgesia [29,51]. This is particularly important as gabapentinoids and opiates such as morphine, which are commonly prescribed for clinical CIPN, alter the motor function of CIPN patients already at increased risk of falls [52,53]. The side effect profile and chemical properties of these standardly used analgesics also did not lend to including them in a prophylactic trial as we designed for EC5026. Opioids at this study duration would cause hyperlocomotion, tolerance and dependence [54,55], and pregabalin would also cause somnolence and gaiting alterations [56,57]. The advantage of the EC5026 was that it could be formulated for delivery in drinking water, which provided good exposure compared to duloxetine used clinically to treat CIPN patients, which does not have adequate water solubility for this delivery [58]. Alternate routes of administration such as daily i.p. injection or oral gavage over 5 weeks of duration would have significant risks of injury to animals already in an induced pain state and were not pursued because the handling stress would change behavioral outcomes separate from the noted side effect changes that would also occur. We have previously investigated sEH inhibition for tolerance, hyperlocomotion and gaiting effects and found that none of these develop in comparison to opioids and gabapentinoids [29]. The monitoring of open-field activity over the entire duration of this experiment demonstrates that there is no evidence of an effect even after 5 weeks of EC5026 drinking water treatment.

The grip strength assay did reveal differences between the groups but, understandably, it was related to docetaxel treatment lowering scores (Figure 3B). EC5026 was not able to affect the decline in grip strength due to docetaxel, but the sEHI-treated docetaxel group did trend better while receiving the weekly chemotherapy injections. All groups including controls received weekly i.p. injections, which may explain the lack of increased scores during the model induction; however, grip strength increased over time with growth after this period, with the controls having higher scores overall.

Growth in weight gain was suppressed with docetaxel administration in both the vehicle- and EC5026-treated docetaxel groups during treatment and did not recover to non-chemotherapy control weight (Figure 4A). Although both groups demonstrated increased weight gain after the docetaxel injections were completed, EC5026 at 1 mg/kg in drinking water was not able to alter the overall lower weight in docetaxel-treated rats. In addition, the livers of EC5026 control rats had a higher gram weight which, when adjusted for body weight, demonstrated a significant increase compared to vehicle controls in a *t*-test analysis (Figure 4B) but was not significant in an ANOVA comparing all groups. The liver/brain ratio (Figure 4C) also demonstrated a significant difference between groups. The higher liver weight did not have functional consequences per the reported behavioral assays including on the free movement and exploration of the animals quantified with the open-field assay. There were also no observable changes in nociceptive behaviors when compared to vehicle controls. This is likely not mechanistically related to the inhibition of sEH because conditional KO mice with tamoxifen-induced ablation of the sEH protein demonstrate no change in body, brain, or liver weights [59]. Overall, the total n of the groups may be underpowered to adequately represent if this increased liver weight observation will persist, but it is worth monitoring in future studies.

At the completion of the behavioral experiments, the observed efficacy was characterized by examining changes in sEH protein concentrations in the CIPN model. There was not an increase in sEH concentrations in the tissues examined due to the docetaxel CIPN model at the end of the 7th week in these experiments (Figure 5). This contrasts with other reports that suggest sEH is increased in select brain regions in mice dosed with systemic LPS or single social stress assessed with semiquantitative Western blot analysis; however, the same study also observed decreased sEH protein in mice from a repeated social stress model using the same methods [60]. In this same study, the postmortem parietal cortex of humans with major psychiatric disorder demonstrated increased sEH [60], and these results were repeated and correlated with the livers, showing similar increases in sEH by Western blot [61]. No other brain region increases in sEH protein or alternative methods were reported in these studies; however, sEH was increased in the striatum of patients with dementia with Lewy bodies compared to controls in another study, again measured by Western blot [62]. An increase in sEH protein in the liver was observed using quantitative real-time polymerase chain reaction (qt-PCR) late in a murine model of chronic mild stress, but there was no increase in prefrontal cortex, hippocampus, or other tissues examined from the same animals [59]. Recently, sEH gene expression demonstrated decreases in expression in midbrain pons and medulla after injury in a model of traumatic axonal injury in the brainstem [63]. The sEH protein has been observed to be moderately increased in other non-neurodegenerative disorders such as alcohol-associated liver disease (ALD) measured by Western blot and qt-PCR for sEH in murine livers [64]. Thus, the outcomes are varied when assessing sEH protein and message. This may be related to the tissue type, species or pathological conditions modeled in these experiments but further characterization and quantification of sEH protein will be required.

A quantitative ELISA was used for the determination of sEH protein to assess the whole brain, spinal cord, sciatic nerve and liver in this rat model of CIPN. As discussed, the liver, whole brain and sciatic nerve data (Figure 5) did not show any increases in sEH protein correlated with the nociceptive sensitivity that was still present at week 7 in the docetaxel controls. It is also notable that the relative amount of sEH protein in the brain here was only 4% of the liver, which is understood to hold the highest concentration of any organ [65]. This is even more impactful when considering the EC5026 control group had a significantly larger liver than other groups but did not exhibit a correlative increase in sEH protein. The spinal cord samples showed a significant increase in the sEH protein level only in the EC5026 + docetaxel group compared to the docetaxel control. The meaning and/or impact of this change is not well understood, especially as it is in contrast to the lack of change in the other nervous system tissues and liver. When considering the overall distribution of sEH expression, there is about 5.5% in the spinal cord compared to the liver and, moreover, the amount of combined brain subregions is equivocal to the amount measured in the choroid plexus ([66] and the Human Protein Atlas website: proteinatlas.org (accessed on 18 April 2025). Thus, there may be a larger role played by the spinal cord and the blood vessels at the barrier between the blood and cerebral spinal fluid. However, the antinociceptive action of sEH inhibition is not known to be exclusive to central or spinal cord-mediated mechanisms. The action of the sEH inhibitor may be mediated in the periphery, with several models demonstrating analgesia [13,18], with an estimated 7–10% brain penetrance for small-molecule sEH inhibitors [60,67]. Additionally, increases in sEH in pathological conditions have been observed in those with underlying inflammatory conditions. Importantly, the samples from these CIPN experiments were taken several weeks after the last docetaxel injection and the return to normal drinking water. Therefore, inflammation may not be at the same level as during the chemotherapy cycles. Further investigation to assess the role of the sEH enzyme, inflammatory markers, and/or microglia activation in the spinal cord of rats with CIPN is warranted.

**Figure A3:**
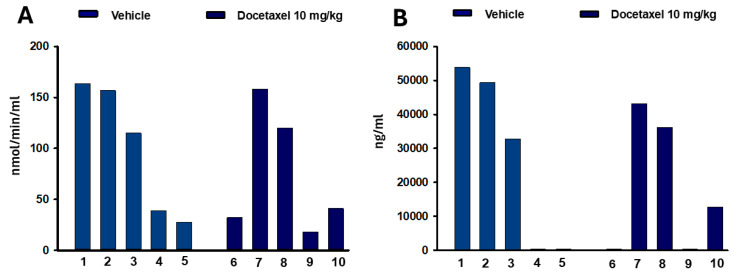
Verification of liver ELISA results with sEH activity assay in a subset of samples. The ELISA (Figure 5) demonstrated that some rats had samples that were below the LOD even for liver tissue which is the highest expression tissue for the sEH enzyme. Therefore, we selected a targeted set of samples including both high concentration and low (under LOD) concentration from vehicle and docetaxel control groups to run in a sEH activity assay. (**A**) The sEH activity assay resulted in minimal activity in the same animals that were low in the original ELISA analysis. (**B**) The ELISA results which were completed first, for the same included samples matching the sEH activity results. The data in both figures is depicted as the same individual samples analyzed per each method and therefore have no SEM. These results confirmed that the differential levels from the ELISA were repeatable and indicated a small number of rats that expressed low amounts of the sEH enzyme.

The sEH protein concentration assessed with the ELISA and a small number of animals in the study fell below the LOD of the assay; however, this change in n/group did not affect the relative concentration between the groups for comparison and so did not appear to bias the outcome. The rats in these groups were not genotyped to determine if a single-nucleotide polymorphism (SNP) or haplotype existed in the Sprague Dawley strain used in the experiments. There are reports of spontaneously hypertensive rats of the Heidelberg SP substrain and Wistar-Kyoto rats having polymorphism in the EPHX2 gene and decreased expression of sEH. There are also several known SNPs in human populations, and some have correlation to disease outcomes [68,69,70,71]. The sEH SNP R287Q, which decreases sEH activity and has protective effects, and K55R having increased sEH activity and deleterious effects, are exonic SNPs known to affect measurable outcomes in human renal function [72]. The effects of the intronic SNP rs6558004 are less understood and the mutation can occur in combination with exonic SNPs [72]. Because the SNPs do not alter the sEH enzyme selectivity for a series of substrates, it was suggested that there is no extra susceptibility to sEH inhibitors in the human population [73]; despite this, the lack of sEH activity with some of the human SNPs remains relevant. The behavioral data from the EC5026-treated docetaxel group, when adjusted for the 2/10 animals that had very low sEH activity, had an improved outcome for the group. Because the sEH activity assay (Figure A3) confirmed substantially lower sEH activity in these animals and removing them did not alter the relative relationship between the groups, the scores were removed from the group analysis and the updated groups (n = 6–8 group) are reported in Figure 5. This was an unexpected outcome in SD male rats, and because the long-term hepatic effects of EC5026 remain to be determined, it is worth further investigation.

Another important consideration is the concern that compounds that alter the development of CIPN may affect the antitumor efficacy of chemotherapeutic agents, in terms of drug–drug interactions or other physiological mechanisms. The cytochrome P450 family of enzymes is important in drug metabolism, with CYP3A4 being involved in metabolizing up to 50% of clinically used drugs including chemotherapeutics and docetaxel specifically [74]. There has also been a meta-analysis of gene variation of CYP3A4 to examine susceptibility to taxane-induced CIPN in humans; however, the investigation found there was not a significant relationship between them [75]. The CYP3A4 enzyme was included in a screening for the effects of EC5026 on metabolism and there was no significant induction or inhibition [28]. Thus, preliminary evidence suggests there is not an interaction or potential for interaction between EC5026 and chemotherapy. Furthermore, the mechanistic differences between sEH inhibition (blocking ER stress and mitochondrial damage [21,23], which supports neuronal health) and docetaxel-induced microtubule stabilization (leading to cell cycle arrest in tumor cells [9]) confers the possibility that the anti-tumor efficacy of the taxane will not be altered. Based on the success of the experimental paradigm in this study, it will be essential to characterize the effects in a tumor-bearing CIPN model that can also determine the antineoplastic efficacy of docetaxel with EC5026 coadministration.

There are limitations to this study including the lack of assessing ER stress markers in this specific docetaxel-induced model of painful CIPN. We also did not assess immune markers or cytokines and focused on behavioral outcomes for our initial investigation of this prophylactic dosing paradigm. The results with organ weight gain in the sEHI controls were not anticipated and this should be the subject of further investigation. In addition, the rats did not bear tumors, and docetaxel was used as a model drug in these otherwise naïve animals. The lack of tumor bearing is often seen as a limitation in painful CIPN preclinical experiments because they lack the tumor microenvironment which is thought to potentially impact neuropathic responses. However, a recent study addressed this concern with orthotopic mouse models of colon cancer and demonstrated that the tumors did not change neuronal damage or macrophage marker intensities and importantly did not find increased pain sensitivity in abdominal mechanical sensitivity, von Frey, or hind paw cold sensitivity for 4 weeks of the testing period [76]. Another concern is that the analgesic agents may interfere with the antineoplastic activity of the chemotherapeutics. With this concern, EC5026 was previously tested in an ADCC assay which evaluated it in combination with Herceptin in a breast cancer cell line looking at natural killer cells as the effector cells. This assay demonstrated that the sEH inhibitor did not alter the action of the chemotherapy despite the known anti-inflammatory (and thus immunomodulatory) activity of this class of compounds [13].

The critique of a non-tumor-bearing preclinical models of CIPN is understandable; however, the neurotoxicity arises from the chemotherapy itself [77]. Across several chemotherapeutic agents that induce CIPN in clinical practice, there is a dose load threshold correlated with CIPN symptom severity which supports this idea [78,79]. CIPN also occurs in post-surgical cases employing adjuvant chemotherapy where there is no known tumor burden (i.e., the tumors have been removed) as well as treatments targeting tumor reduction. Additionally, a large portion of patients treated at or above the cumulative dose loads have persistent painful neuropathy for months into their recovery from cancer (again, remission with no, or no sizable, tumor load). Thus, although testing in a tumor-bearing model is a rational next step, the results from examining the efficacy of EC5026 in a tumor-free CIPN model remain valuable.

## 4. Materials and Methods

### 4.1. Animals

All included experiments were conducted in accordance with protocols approved by the Institutional Animal Care and Use Committee of the University of California and adhered to the National Institutes of Health guide for the care and use of laboratory animals. Great care was taken to reduce the number and minimize the suffering of the animals used. Sprague–Dawley male rats (250 to 300 g; Charles River, Wilmington, MA, USA) were housed on corncob bedding (2 per cage) with free access to food and water. They were maintained under a 12 h light/dark cycle with controlled temperature and relative humidity. Randomization and blinding: The preclinical studies used stratified randomization conducted after the acclimation and baseline assessment of rodents. This allowed for block randomization of sample size and balancing of the disease state covariate for assessing the therapeutic treatments. We also employed allocation concealment with the behavioral assay observers blinded to the group identification of the individual animals as well as the treatment they received. Testing of assays was performed between 8:00 a.m. and 5:00 p.m. at the same time of day and on the same day per week throughout the course of the experimental design.

### 4.2. Chemicals

The sEHI EC5026 was synthesized per previously reported methods [28] and formulated in PEG400 (Sigma Aldrich, St. Louis, MO, USA). The compound or vehicle was administered by formulated drinking water consisting of 1% PEG400 in water. Commercial docetaxel formulated for human use was used as the chemotherapeutic agent (Fisher Scientific, Pittsburgh, PA, USA) for this study and was administered as a 10 mg/kg i.p. dose injected once weekly for three weeks. To avoid acute necrotic effects with an intraperitoneal injection of the 20 mg/mL stock formulation, the docetaxel solution was diluted 10-fold immediately prior to injections with sterile saline. The injected volumes ranged from 1.5 to 2.1 mL per body weight using a 2 mg/mL final solution of docetaxel. The male rats were then returned to their home cages and monitored daily for three days before any additional assessment each week.

### 4.3. Blood Concentration Determination

Two independent experiments were run to characterize the pharmacokinetics of EC5026 in rats. First, a subset of the animals receiving drinking water formulation and undergoing the behavioral assays were sampled to monitor sEH inhibitor levels. For this, 10 uL of whole blood was sampled from the tail vein via venipuncture and was added to 90 uL DI water containing 0.1% EDTA. These samples were analyzed with LC-MS/MS per the following methods. Samples were analyzed by multiple reaction-monitoring modes on the Xevo TQS mass spectrometer (Waters, Milford, MA, USA) and referenced to an internal standard (d5-EC5026). The optimized parameters of mass spectrometers for monitoring this sEH inhibitor are described as follows: transition from first quadrupole to third quadrupole was 404.2 to 194.0 mass-to-charge ratio, capillary voltage was −3 KV, cone voltage was −20 V, collision energy was −28 V, and LOD was 0.1 ng/mL.

Second, because the experimental design of the prophylactic administration of EC5026 in drinking water to docetaxel-treated rats did not allow for assessing the acute pharmacological interaction of the two compounds, we acutely administered the two compounds in a satellite group of male rats. This allowed us to investigate potential drug–drug interactions that would alter the blood concentration of docetaxel. Repeated oral gavage administration of EC5026 (daily for four consecutive days) prior to one dose of docetaxel was used and followed with sampling whole blood for mass spectrometry analysis and quantification. For this, we first sampled a pretreatment blood sample and then orally gavaged the animals once a day at the same time of day with a 1 mg/kg dose of EC5026 or vehicle, both in 1 mL volume. On the fourth day of oral gavage, a blood sample was taken 1 h after gavage of EC5026 or vehicle and then docetaxel 10 mg/kg was injected i.p. to all rats and 10 uL whole blood samples from the tail vein were taken serially as follows: at 15 and 30 min and at 1, 1.5, 2, 2.5, 3, 4, 5 and 6 h. The samples were frozen at −20 °C until mass spectrometry analysis. The results are depicted in Figure 2C.

### 4.4. Behavioral Assessment and Husbandry

Cage changes occurred on Monday of every week with a measured body weight but no behavioral assays. The rats were given 24 h in new caging before the first assay was performed on the next day. For all assays including baseline assessment, the rats were tested at the same time of day to minimize the impact of circadian variations. Standardized handling procedures and a consistent environment including temperature, light and noise level were maintained throughout the entire duration of the experiments. Open-field assays were conducted once a week on the same day and at the same time of day but with groups of rats in a balanced rotation during the experiment. The von Frey assay was conducted on the subsequent 3rd day of the week, again always in the same weekly sequence and same time of day, but with a balanced rotation of groups. The grip strength and cold plate assays were conducted on the next subsequent day at the same time of day and with balanced rotation. Groups were assayed first in the grip strength test until all groups were completed and returned to home cages; then, the groups were tested in the cold plate assay with the same rotation as the grip strength assay. On the 5th consecutive day, blood collection of a subset of all groups for pharmacokinetic (PK) analysis was conducted starting at time of lights on until completion and with a balanced rotation weekly for the duration of the study.

### 4.5. Experimental Design

**Figure 6:**
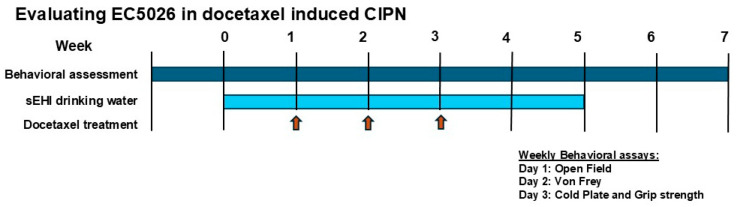
Experimental design of prophylactic sEH inhibitor (EC5026) administration in painful docetaxel CIPN.

The nociceptive and functional assays were conducted after acclimation and pre-exposure to testing equipment to assess baseline scores for naïve animals to assess individual differences prior to treatment (Figure 6). The rats then began formulated drinking water treatment, either 1% PEG vehicle or EC5026 in 1% PEG drinking water. The same weekly routine of assays was performed to assess any possible effect of the drinking water treatments compared to naïve baselines. The next week, while continuing drinking water treatments and routine behavioral assays, the rats were treated with the first of three total doses of docetaxel 10 mg/kg or saline (for controls) i.p. injections. The behavioral assay routine continued through all three weekly doses of docetaxel or saline. The rats were then assayed for an additional week, and after the behavioral assay schedule was complete, all groups including controls were changed to normal drinking water to observe the effects on recovery. The rats continued to be assayed on the same schedule for 2 weeks on regular drinking water. At the end of these 2 weeks (7th total week of experiment), the animals were euthanized and sampled for tissues.

### 4.6. Nociceptive and Functional Assays

Rats were acclimated with pre-exposure to the testing apparatus and tested for baseline scores before the induction of CIPN models. We tested the rats for the development of allodynia using the von Frey assay using an electronic aesthesiometer with a rigid tip to assess mechanical withdrawal thresholds, and rats with confirmed sensitivity were then randomized for treatments. The von Frey scores are 5 trials per rat averaged per animal and reported as the mean ± SEM grams per group to induce a withdrawal response.

We assessed cold pain with a commercial cold plate (IITC, Woodland Hills, CA, USA). For this, rats were placed on a cold plate, and the time taken for the first brisk lift or stamp of the hind paw to occur was recorded. The cold plate was set at 5 °C; at this temperature, a thin film of cold condensation is visible on the plate, which was wiped clean before the animal entered the chamber. A cutoff of 15 s was used for 5 °C. The animals were given a minimum 3-min recovery time before any repeat testing and no more than three trials for any time point were conducted. Scores are reported as the mean ± SEM latency in seconds per group.

Docetaxel in humans has been reported to involve motor neuropathy in addition to sensory neuropathy [35]. Due to this and possible changes in activity due to the pleiotropic effects of chemotherapy treatments on overall health, we assessed the open-field activity of all rats in the study as well as using a grip strength assay to evaluate weakness [11] throughout the entire duration of the experiment. We assessed exploration and motor skills in naïve rats with the open-field assay before induction of the CIPN models, and then weekly for the duration of the experiment including the transition to normal drinking water at the end. For the open-field assay, rats were placed in an open-field arena (40W × 40L × 30H cm) of a 16-square-grid clear-acrylic open-top chamber and observed for 2 min continuously. Activity was assessed and scored as the sum of lines each animal crossed with both hind paws and number of rears as a function of time. The grip strength assay used a metal grid square (both commercial equipment from IITC Inc., Woodland Hills, CA, USA) connected to an electronic read-out meter that displays grams of force. In each case, the rat was allowed to grasp the grid and was pulled away gently from the apparatus by the tail, and the grams of force of their grip strength while being pulled away was recorded. The rats were tested with three trials per rat with a 1 min interval between trials, and the score was averaged per rat and reported as the mean ± SEM grams per group.

### 4.7. sEH ELISA Protein Quantification

The tissues were sampled after the end of tahe behavioral study and snap-frozen at −80 °C until processing. The samples were thawed and homogenized in protease-containing buffer and diluted before assaying on a 96-well plate for enzyme-linked immunosorbent assay (ELISA) to quantify the sEH enzyme per published methods [80].

**Figure 7:**
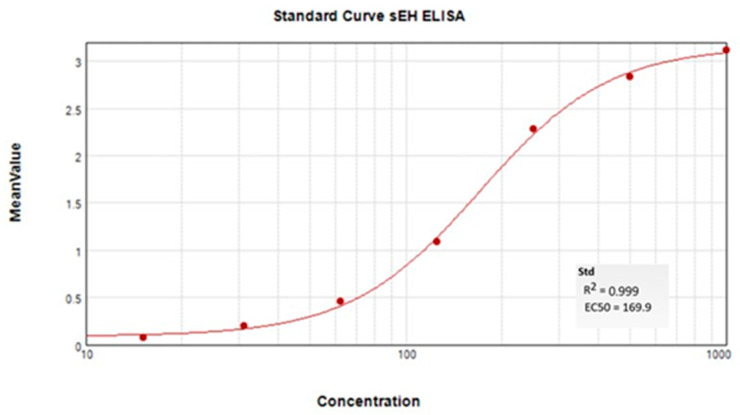
Representative standard curve of sEH ELISA analysis.

Despite appropriate standard curves run on every 96-well plate (Figure 7, example curve from sample set), the assay resulted in lower-than-LOD measurements for some samples in each of the n = 10 groups. A subset of these low-concentration samples and some higher-concentration samples were assessed with an sEH activity assay, which confirmed the validity of the results. The samples with lower values than the LOD were not included in the group average (depicted in Figure 5), and this change in n/group did not affect the relative concentration between the groups for comparison. The remaining n/group (n = 6 for docetaxel and EC5026 groups, n = 8 for vehicle and EC5026 + docetaxel groups) is reported in the Results Section with information about the statistical analysis.

### 4.8. Statistics

Statistics were computed using Systat Software Inc. Sigmaplot 14.0 for Windows (San Jose, CA, USA). All results are expressed as mean ± standard error of the mean (SEM) for groups of male rats (n = 10/group) that were statistically powered to detect significant differences between independent groups, with averages as primary endpoints. Parametric data were analyzed with analysis of variance with the specific post hoc methods indicated in each case. Nonparametric data were analyzed using ranked methods and all data with repeated measurements were analyzed with an appropriate repeated-measure statistical test, with *p* <  0.05 considered significant for all tests.

## 5. Conclusions

These experiments had the intent of assessing the impact of prophylactic administration of EC5026 in a docetaxel-specific CIPN model. The results demonstrated the efficacy of EC5026 against different pain modalities and the promise of limiting a notoriously intractable chronic pain condition.

## Data Availability

The original contributions presented in this study are included in the article. Further inquiries can be directed to the corresponding author(s). The raw data supporting the conclusions of this article will be made available by the authors on request.

## Abbreviations

The following abbreviations are used in this manuscript:

CIPN	Chemotherapy-induced peripheral neuropathy
CNS	Central nervous system
sEH	Soluble epoxide hydrolase
sEHI	Soluble epoxide hydrolase inhibitor

## Appendix A

**Table A1:** sEH inhibitor potency on recombinant rat and mouse sEH enzyme.

	IC50 (nM)	
	Rat sEH	Mouse sEH
TPPU	7.6	5.0
EC5026	1.4	1.6

### Measurement of Inhibitor Potencies on Rat and Mouse sEH

The recombinant mouse and rat sEH enzymes were expressed and affinity-purified, and the reported IC50 values were obtained using fluorescence-based assays [38,39]. In a black 96-well plate, 150 μL of buffer (NaPO4 0.1M ph 7.4 containing 0.1 mg/mL of BSA) was dispensed in all the wells; then, 2 μL of either DMSO (for the 100% activity control wells) or a solution of the inhibitor concentrated 100× in DMSO for the test wells was added (0.4 ≤ [I]final ≤ 50,000 nM). After strong mixing, 20 µL of buffer was added to the blank wells, while 20 µL of 10× enzyme solutions ([E]final ≈ 2 nM for the mouse sEH and ≈ 3 nM for the rat sEH) was added in the 100% activity (no inhibitor) and test wells (with inhibitors). The plates were mixed and incubated at 37 °C for 5 min in the dark. In the meantime, the working solution of substrate (nonfluorescent MNPC: cyano(6-methoxy-naphthalen-2-yl)methyl trans-[(3-phenyloxiran-2-yl)methyl] carbonate) was prepared by mixing 3.22 mL of buffer and 230 μL of stock 0.5 mM MNPC in DMSO. The enzymatic reaction was started by the addition of 30 μL of the working solution of substrate to each well ([S]final = 5 μM). The formation of the fluorescent product (6-methoxynaphthaldehyde) was measured in a microplate reader M2 (Molecular Devices., CA, USA) at 37 °C in kinetic mode for 10 min with measurement every 30 s (λem = 330 nm, λex = 465 nm, λcutoff = 430 nm). All measurements were performed in triplicate. The inhibition of the enzyme was calculated for each [I] based on 100% activity wells (DMSO only). The IC50s are defined as the concentration of inhibitors that reduce the activity by 50% and were calculated by including at least two datum points above and two datum points below the IC50. High Z′-factor values (0.75–0.80) were obtained for the assay. The standard error for the IC50 values is between 10% and 20% for the assays performed here, suggesting that only differences of two-fold or greater would be significant.

Painful CIPN was induced with three weekly doses of 10 mg/kg docetaxel i.p. (the same induction regimen used for the prophylactic treatment described in the main text). After the painful CIPN was established and was tested for baseline pain with the von Frey assay, the rats were orally gavaged with EC5026 at the doses indicated and followed over a time course. The von Frey assay results demonstrate efficacious and dose-dependent analgesia in the docetaxel-induced CIPN model (two-way repeated-measure ANOVA, Holm–Sidak method post hoc analysis compared to vehicle, n = 5–8/group).

All groups in the experiment were provided with 1% PEG400 formulated drinking water which was the vehicle for EC5026. All groups receiving EC5026 or vehicle were administered formulated drinking water 5 weeks before being returned to regular drinking water for 2 additional weeks. The drinking water for all groups was served ad libitum in clean and sterilized acrylic bottles with animals housed at two per cage. The drinking water bottles were weighed at the start and end of each cycle to monitor consumption. The total milliliters (mL) per time period was divided by the number of days the water was provided, and the total weight of both animals was used to calculate the mL per gram body weight ratio depicted in the figure. While this is only an estimate, this measurement documented the general consumption over the course of the experiment and provided a way to monitor any unexpected leaks or decreases in consumption for all included animals prior to the actual mass spectrometry analysis of the blood samples from selected individual animals. The ratios indicate that formulated drinking water of either vehicle or EC5026 treatments was well tolerated and consumed in expected average amounts. In particular, the vehicle and EC5026 control groups not receiving chemotherapy were well matched and consistent over the experiment. These groups demonstrated a transient decline in ratio at the end of the chemotherapy regimen (days 21–28), but this reflects the increased body weight in the denominator of the ratio compared to docetaxel-treated groups at this stage. There was also a slight decline in consumption with the transition to normal drinking water for all groups (day 42) that could be due to neophobia of the water change, but also may reflect a body weight increase in the denominator as well.
